# Optimal and Robust Design Method for Two-Chip Out-of-Plane Microaccelerometers

**DOI:** 10.3390/s101210524

**Published:** 2010-11-24

**Authors:** Sangmin Lee, Hyoungho Ko, Byoungdoo Choi, Dong-il Dan Cho

**Affiliations:** 1 Automation and Systems Research Institute (ASRI)/Inter-university Semiconductor Research Center (ISRC), School of Electrical Engineering & Computer Sciences, Seoul National University, Seoul, Korea/Daehak-dong, Kwanak-gu, Seoul 151-019, Korea; E-Mails: sangmlee@snu.ac.kr (S.L.); bdchoi1@snu.ac.kr (B.C.); 2 Department of Electronic Engineering, Chungnam National University, Gung-dong, Yusung-gu, Daejeon 305-764, Korea; E-Mail: hhko@cnu.ac.kr

**Keywords:** design optimization, out-of-plane microaccelerometer, Extended Sacrificial Bulk Micromachining (ESBM) process, CMOS capacitive readout circuit

## Abstract

In this paper, an optimal and robust design method to implement a two-chip out-of-plane microaccelerometer system is presented. The two-chip microsystem consists of a MEMS chip for sensing the external acceleration and a CMOS chip for signal processing. An optimized design method to determine the device thickness, the sacrificial gap, and the vertical gap length of the M EMS sensing element is applied to minimize the fundamental noise level and also to achieve the robustness to the fabrication variations. In order to cancel out the offset and gain variations due to parasitic capacitances and process variations, a digitally trimmable architecture consisting of an 11 bit capacitor array is adopted in the analog front-end of the CMOS capacitive readout circuit. The out-of-plane microaccelerometer has the scale factor of 372 mV/g∼389 mV/g, the output nonlinearity of 0.43% FSO∼0.60% FSO, the input range of ±2 g and a bias instability of 122 μg∼229 μg. The signal-to-noise ratio and the noise equivalent resolution are measured to be 74.00 dB∼75.23 dB and 180 μg/rtHz∼190 μg/rtHz, respectively. The in-plane cross-axis sensitivities are measured to be 1.1%∼1.9% and 0.3%∼0.7% of the out-of-plane sensitivity, respectively. The results show that the optimal and robust design method for the MEMS sensing element and the highly trimmable capacity of the CMOS capacitive readout circuit are suitable to enhance the die-to-die uniformity of the packaged microsystem, without compromising the performance characteristics.

## 1. Introduction

Over the last decade, extensive efforts have been devoted to the continuously maturing Microelectromechanical System (MEMS) technologies. Above all, MEMS microaccelerometers have been successfully commercialized in a wide range of application areas including automotive safety control, ubiquitous robots, inertial navigation and consumer electronics [1,2]. Most of these applications require sensors which have a multi-axial operation, high sensitivity, large dynamic range, low noise floor and low bias instability, while maintaining low-cost and mass-productivity. The current commercial-off-the-shelf (COTS) MEMS accelerometers have multi-axial operation, while maintaining the noise-floor between a few tens to hundreds μg level. However, the noise-level of the out-of-plane axis is generally higher than that of the in-plane axes. In order to improve the noise performance of the out-of-plane axis, an optimized design method considering the mechanical-thermal noise limit of the MEMS sensing element should be applied.

Recently, a microsystem using a two-chip solution consisting of a MEMS element and a CMOS readout circuit has been implemented so as to improve the noise performance [3–5]. A specialized MEMS bulk micromachining process and an advancement of packaging technology have been established to enable a miniaturized microsystem, while maintaining a low fundamental noise level. Moreover, an optimal and robust design of the MEMS sensing element is necessary so as to enhance the robustness to the fabrication variations. Amini *et al.* [3] have demonstrated an in-plane micro-gravity accelerometer by optimizing the gap size of comb electrode. Ko *et al.* [4] presented a design principle of an in-plane accelerometer to optimize the thickness of device layer to minimize the fundamental noise limit. In these papers, however, the dimensional optimization is limited for an in-plane accelerometer and performance variation due to process variation is not discussed. For the low noise characteristic of an out-of-plane operation, Hsu *et al.* [5] calculated the estimated thermal-mechanical noise. However, this paper only acknowledged the designed result, and did not deal with the design procedure to minimize the noise floor in detail.

The capacitive sensing scheme provides advantages in low temperature dependency, good DC response, and good noise performance [6]. However, the performance of a capacitive sensing sensor is severely limited by the parasitic capacitance. In case of two-chip implemented microsystem, the parasitic capacitances randomly exist mainly due to process variations, bonding wire and bonding pad, which is in several pF ranges [7,8]. Several research groups have reported the method to compensate the process variations using capacitive readout ICs [3,9,10], but the trimmable range for compensation is limited. Therefore, to determine the compensation capability of the CMOS capacitive readout circuit, the capacitance variation of the MEMS sensing element due to fabrication process should be analyzed along with the implementation of a highly trimmable architecture.

In this paper, an implementation of an out-of-plane microaccelerometer system employing an optimal and robust design method that achieves robustness towards the fabrication variations and enhances the die-to-die uniformity without compromising the performance characteristics is presented. The optimal design method is based on the minimization of the total noise equivalent acceleration (TNEA) of the two-chip implemented microsystem. Besides lateral dimensions such as width and length of the torsional spring and gap between the comb electrodes, vertical dimensions such as structural thickness and sacrificial gap of the sensing element and vertical gap length between the moving and stationary vertical comb electrode are taken into consideration for the several reasons, which are discussed later in this paper. The sensor operation is based on a coplanar sense electrode movement wherein the change in capacitance is caused by variation of the overlap area [11] rather than in the air gap [12]. This differential sensing scheme enables the design of a wide dynamic range out-of-plane accelerometer. Another advantage of this sensing scheme is that squeeze film damping between the movable proof mass and the substrate can be minimized by fabricating a large sacrificial gap. Since, the proposed microaccelerometer adopts a CMOS and MEMS, two-chip packaged implementation, the mechanical damping of the MEMS sensing element can be an important issue when demonstrating a low noise device. The out-of-plane microaccelerometer is fabricated by the Extend Sacrificial Bulk Micromachining (ESBM) process [13] and wafer-level hermetic packaging (WLHP) process [4]. The ESBM process is a simple, two-mask fabrication process, which is able to fabricate a high-aspect-ratio structure with a large sacrificial gap and to fabricate the upper and lower vertical gap between the interdigitated comb electrodes.

The brief features mentioned above will be described in the following sections. Beginning with a concept of a two-chip implemented microsystem, the optimal design analysis to determine the device thickness and the vertical gap length will be followed. The design will be substantiated by both electrostatic and mechanical analysis as well as finite element method (FEM) simulation. Then, the advantages of the separate two-chip implemented microsystem will be discussed. After the fabrication principles and fabrication results, the experimental results are evaluated. Finally, conclusions will be drawn.

## 2. Two-Chip Implemented Microsystem

In this microaccelerometer system, a two-chip solution comprising of separated CMOS sensing electronics and MEMS element is adopted. This two-chip solution allows specialized and optimized processing for CMOS and MEMS [14]. However, with the two-chip implementation compared to the monolithic integration, the dynamic range and the gain of the microsystem can be limited due to increased parasitic capacitance [15]. In order to cancel out the offset and gain variations due to parasitic capacitances and to minimize the die-to-die variation due to process variations, a digitally trimmable architecture consisting of capacitor arrays is adopted in the CMOS capacitive readout circuit [16].

**Figure 1.**
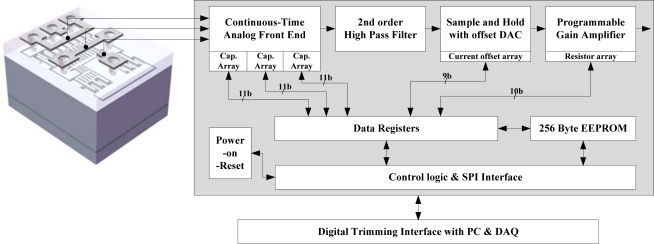
Top level block diagram of the two-chip implemented microsystem.

In Figure 1, the top level block diagram of the two-chip implemented microsystem is shown. The MEMS sensing element is fabricated by the ESBM process and the WLHP process. The capacitance change of the MEMS sensing element is converted to a modulated voltage signal by the continuous-time front-end charge amplifier. The compensation of offset and gain variations is performed using 11 bit programmable capacitor arrays. The low frequency noise components of the modulated signal are attenuated by the following high pass filter. Then, the modulated voltage signal is demodulated by a sample-and-hold demodulator and offset calibration of the signal is performed in this stage using a 9-bit current-mode digital-to-analog converter (DAC). Next, the unnecessary high frequency noise components are eliminated by a low pass filter, and the desired signal is obtained. The gain of the signal can be calibrated using a 10 bit programmable gain amplifier (PGA). The programmed data is stored to the 256 byte EEPROM block, and reloaded to the registers when the power is turned on.

## 3. MEMS Sensing Element Design

**Figure 2.**
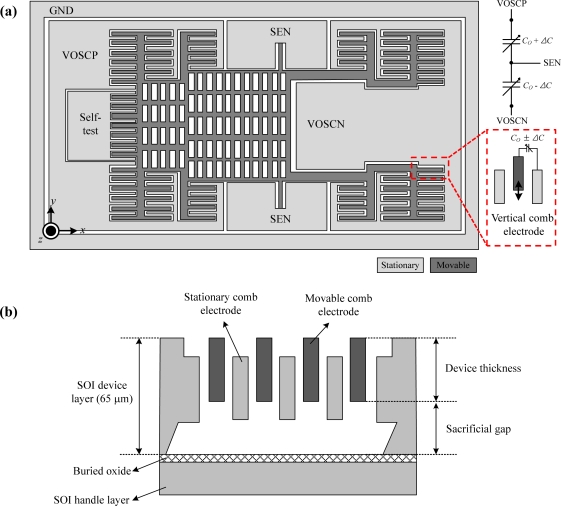
**(a)** The planar view of conceptual schematic. **(b)** The cross-sectional. The conceptual schematic diagram of a capacitive out-of-plane torsional microaccelerometer.

The conceptual schematic diagram of a capacitive out-of-plane torsional microaccelerometer is illustrated in Figures 2(a,b). The sensing element is designed to have an asymmetric proof mass suspended by two guided-end torsional springs and comb electrodes. In addition, stationary comb electrodes and movable comb electrodes form an interdigitated pair to detect capacitance change. In this MEMS sensing element design, the differential capacitive sensing scheme is employed. A vertical gap is formed between stationary comb electrodes and movable comb electrodes in upper and lower parts, so as to enhance the mechanical sensitivity, linearity and noise performance. When external acceleration is applied, the guided-end springs are twisted by torque acting on the asymmetric proof mass of the sensing element. The rotation of this asymmetric mass makes a change in the overlap area between the stationary and movable comb fingers, which results in an increment or a decrement of the capacitance.

In this paper, the lateral dimensions are fixed and only the vertical dimensions, which is determined by the fabrication process is taken into consideration. As the gap between the comb electrodes is increased, the mechanical damping gets smaller and the Brownian noise equivalent acceleration (BNEA) is improved. However, the mechanical sensitivity, which is determined by the capacitance change between the interdigitated comb electrodes, is decreased and the circuit noise equivalent acceleration (CNEA) is increased. Therefore the minimum gap size of 4 μm is chosen to maximize the mechanical sensitivity. The torsional spring stiffness is determined by considering the input dynamic range of ±2 g. As the torsional spring stiffness is decreased, the noise performance is enhanced due to the decrement of the 1st order natural frequency and also mechanical sensitivity can be improved. However, the process yield during the fabrication process and the impact resistance of the sensing element get poor due to the decreased torsional stiffness [17,18]. For this reason, the mask dimension of the spring width and length is determined to be 4 μm and 150 μm, respectively. Therefore the proposed design method is applied to optimize the vertical dimensions of the MEMS sensing element, so as to minimize the noise level and also to achieve the robustness to the fabrication variations.

### 3.1. Optimization of Device Thickness

**Figure 3.**
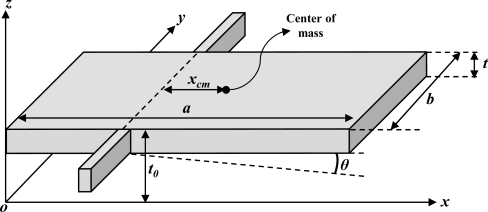
A Simplified schematic of the torsional microaccelerometer.

The design parameters of the MEMS sensing element are listed in Table 1. In order to maximize the performance characteristic and the noise performance, it is necessary to determine the optimal structural thickness which minimizes the mechanical-thermal noise. The fundamental sense limit of the MEMS sensing element is set by the BNEA of the suspended proof mass [19]. The BNEA is expressed as:

(1) $$\text{BNEA}=\sqrt{\frac{4k_{B}T\omega_{n}}{\text{mQ}}}\left[m/s^{2}/Hz^{1/2}\right]$$

where *k_B_* is the Boltzmann constant (1.38 × 10^−23^ m^2^kg/s^2^/K), *T* is the absolute room temperature (300 K), *ω_n_* is the 1st mode natural frequency of the microacceleromter, *m* is the mass, and *Q* is the mechanical quality factor. For an analysis of the mechanical quality factor, the squeeze film damping model [20,21] is applied between movable proof mass and substrate. According to [22], the Stoke’s flow damping between the interdigitated comb electrodes is negligible compared to the squeeze film damping between two plates. The sensing element is designed to be operated at an atmospheric pressure to avoid the resonance and the unstable transient response. A simplified schematic of torsional out-of-plane microaccelerometer is shown in Figure 3.

**Figure 4.**
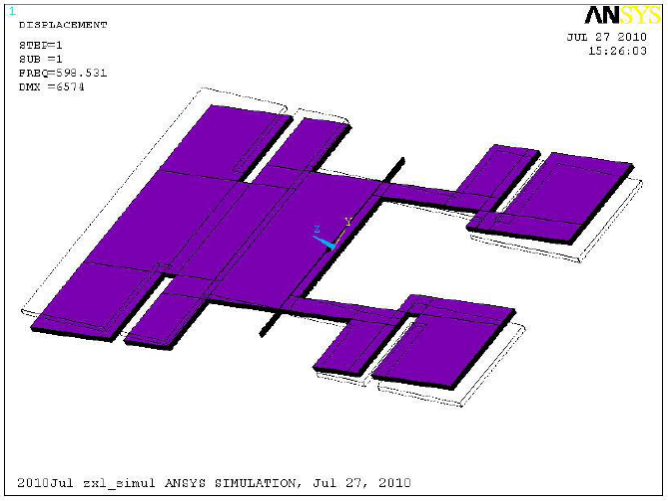
Modal analysis result of 1st order natural frequency.

The squeeze film damping coefficient of the two parallel plates [23] is derived as:

(2) $$\xi =\frac{1152a^{4}\mu_{\text{eff}}}{\pi^{4}{\left(t_{0}-t\right)}^{3}\rho_{s}t\left(a^{2}+t^{2}\right)\omega_{n}}\Delta_{nm}$$

(3) $$\Delta_{\text{nm}}=\sum_{n=1,2\ldots }^{\infty }\sum_{m=1,2\ldots }^{\infty }\frac{1}{{\left(2n\right)}^{2}{\left(2m-1\right)}^{2}}\times \frac{1}{{\left(2n\pi \right)}^{2}+{\left(\left(2m-1\right)\pi \frac{a}{b}\right)}^{2}}$$

where *μ_eff_*, *t_0_*, *t*, *ρ_s_*, *ω_n_*, *a* and *b* represent the effective coefficient of air viscosity (1.98 × 10^−5^ Ns/m^2^), the device layer thickness of silicon-on-insulator (SOI) wafer (65 μm), the structural layer thickness of the sensing element, the density of the silicon (2,330 kg/m^3^), the natural frequency of the MEMS sensing element [598 Hz (Figure 4)], the length of the device, and the width of the device, respectively.

For the case where t << a, the quality factor is given as:

(4) $$Q=\frac{1}{2\xi }=\frac{\pi^{4}{\left(t_{0}-t\right)}^{3}\rho_{s}t\left(a^{2}+t^{2}\right)\omega_{n}}{2304a^{4}\mu_{\text{eff}}\Delta_{\text{nm}}}\approx \frac{\pi^{4}{\left(t_{0}-t\right)}^{3}\rho_{s}t\omega_{n}}{2304a^{2}\mu_{\text{eff}}\Delta_{\text{nm}}}$$

Since the device layer of the SOI wafer has a thickness limit of 65 μm, the sacrificial gap between two plates decreases as the device thickness increases, as illustrated in Figure 2(b). This implies that to minimize the BNEA level, the structural thickness must have limitation at a certain level.

**Figure 5.**
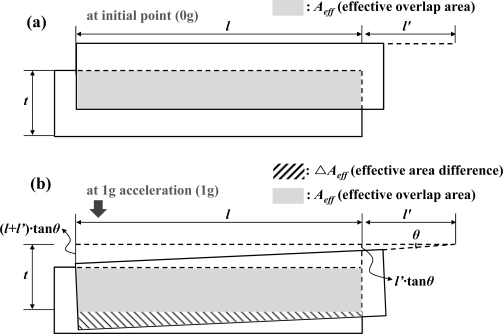
**(a)** Effective overlap area at zero input acceleration. **(b)** Effective overlap area and effective area difference at 1 g input acceleration. Cross-sectional diagram of interdigitated sensing comb electrode.

Another limiting factor is the circuit noise equivalent acceleration (CNEA) that depends on the simulated minimum detectable capacitance of the readout circuit (Δ*C_min_*) and the mechanical sensitivity of the MEMS sensing element (*S* = Δ*C/g*). In Figures 5 (a,b), the cross-sectional schematic of the interdigitated comb electrode is shown. Using the detailed parameters from Table 1, the mechanical sensitivity can be derived. The CNEA and mechanical sensitivity can be expressed as:

(5) $$\text{CNEA}=\frac{\Delta C_{\text{min}}}{S}\left[m/s^{2}/Hz^{1/2}\right]$$

and:

(6) $$S\frac{\Delta C}{g}=\frac{n{ɛ}_{0}}{d}\left\{\frac{l}{2}\left[\left(l+2l^{\prime }\right)\theta \right]\right\}\left[F/g\right]$$

where *n*, *ɛ_0_*, *d*, *l*, *l’* and *θ* is the number of the comb fingers, the permittivity of air (8.854 × 10^−12^ F/m), the gap between the comb fingers, the overlap length between the comb fingers, the effective distance from the center to the comb fingers, and the torsion angle at 1 g (9.8 m/s^2^) input, respectively. As structural thickness is increased, the torsion angle at 1 g input (*θ*) and mechanical sensitivity (*S*) is decreased. The overall noise performance of the microsystem, which consists of the MEMS sensing element and the capacitive interface circuitry, can be analyzed using the TNEA analysis. The TNEA can be calculated as a geometric mean of BNEA and CNEA, *i.e.*:

(7) $$\text{TNEA}=\sqrt{{\text{BNEA}}^{2}+{\text{CNEA}}^{2}}\left[m/s^{2}/Hz^{1/2}\right].$$

**Figure 6.**
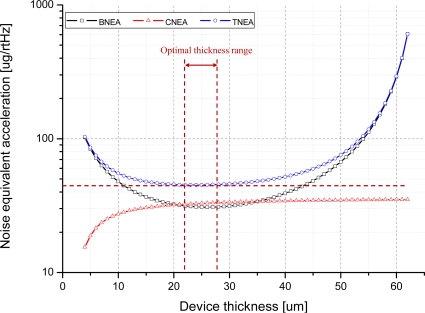
Noise Equivalent Acceleration *vs.* device thickness.

Figure 6 shows the relationship between the device thickness and the BNEA, CNEA, and TNEA. It is known that noise performance is improved as the structural thickness of the sensing element is increased [4]. However, the proposed out-of-plane microaccelerometer is fabricated on a SOI wafer which has a device layer thickness of 65 μm, therefore, as the thickness of the sensing element increases the thickness of the sacrificial layer is decreased, which results in a low mechanical quality factor and a high TNEA level. This result implies that to achieve the minimum TNEA level, the optimized structural thickness is between 23 μm and 27 μm. When the device thickness is 25 μm, the BNEA is 30.79 μg/(Hz)^1/2^, the CNEA for Δ*C_min_* at 0.2 aF/(Hz)^1/2^ is 15.45 μg/(Hz)^1/2^, and the TNEA is 45.06 μg/(Hz)^1/2^.

### 3.2. Optimization of Vertical Gap Length

The proposed out-of-plane microaccelerometer has a vertical gap formed between stationary comb electrodes and movable comb electrodes in the upper and lower parts. In previous studied out-of-plane accelerometers [11,24], a conventional formula of parallel-plate capacitance is used to derive the mechanical sensitivity for the ease of evaluating the device characteristics in analytical expression. The accuracy of the evaluated result can be improved by assuming a constant fringing field method such as Palmer’s formula [25]. These approaches can be valid for a capacitive sensing structure using an air-gap sensing mechanism. However, for a comb-finger typed out-of-plane structure, the effect of fringing field varies due to vertical gap length. Therefore, electrostatic simulation using FEM tool (ANSYS, Inc., United States) is done to verify the optimized vertical gap length.

In order to derive the capacitance, a simplified three dimensional simulation model with two parallel-plates is used. A 10 V (*V_0_*) source is applied to the inner comb electrode and a 1 V (*V_1_*) source is applied to the outer comb electrode. Then, from the FEM simulation the electrostatic energy, *W*, and the nominal capacitance, *C_ANSYS_*, can be calculated using Equation (8):

(8) $$C_{\text{ANSYS}}=\frac{2W}{{\left(V_{0}-V_{1}\right)}^{2}}\left[F\right]$$

**Figure 7.**
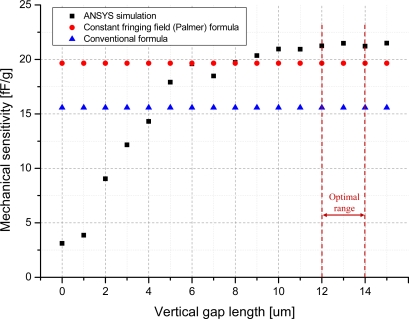
Simulation result of mechanical sensitivity *vs.* vertical gap distance.

The simulation is performed for the zero input acceleration and the 1 g input acceleration to derive the mechanical sensitivity. In this out-of-plane microaccelerometer, due to the dimensional limitation of the design constraints, the vertical gap length is set to have a value below 15 μm. Therefore variation of the vertical gap length is carried out from 0 μm to 15 μm. The analyzed result of the mechanical sensitivity using FEM simulation is plotted in Figure 7.

In order to compare the result, the mechanical sensitivity derived from a conventional parallel-plate formula and a constant fringing capacitance formula is also plotted. The mechanical sensitivity increases due to the increase of the vertical gap length from 0 μm to 10 μm. However, at length between 11 μm and 15 μm, the mechanical sensitivity is in the range from 20.6 fF/g to 21.5 fF/g. Due to the analyzed result and the consideration of fabrication process error about 10%, the vertical gap length is determined to be within the range from 12 μm to 14 μm.

## 4. Capacitive Interface Circuit Design

**Figure 8.**
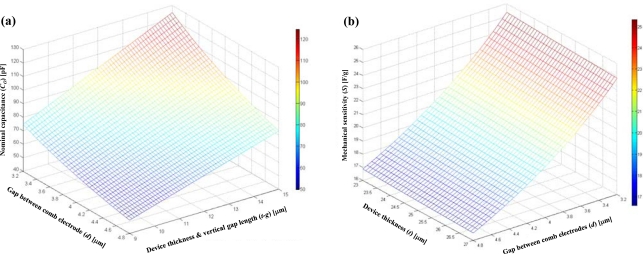
**(a)** Nominal capacitance due to gap size, device thickness and vertical gap variation. **(b)** Mechanical sensitivity due to gap size and device thickness variation. Performance range due to process variation.

Although many advanced micromachining processes are developed, fabrication imperfection is inevitable with current MEMS fabrication techniques [26]. For example, the resonant frequency of a resonator is mostly determined by the spring stiffness, and the practical tolerance in the spring width during the fabrication process is about 10% [27]. Also, the pattern variation due to over-exposure and under-exposure occurs frequently during the photolithography step. The imperfections in the deep reactive ion etching (DRIE) process, such as undercut and footing phenomenon, also results in a large process variation. In this circuit design, the expected variation range of structural thickness (*t*), vertical gap length (*g*), and gap between two parallel comb electrodes (*d*) are from 23 μm to 27 μm, from 12 μm to 14 μm, and 3.2 μm to 4.8 μm, respectively. From Table 1, the variation of nominal capacitance (*C_O_*) and mechanical sensitivity (*S*) can be expressed as:

(9) $$C_{O}={ɛ}_{0}\frac{l\left[\left(t+\Delta t\right)-\left(g+\Delta g\right)\right]}{\left(d+\Delta d\right)}={ɛ}_{0}\frac{l\left[\left(t-g\right)+\left(\Delta t-\Delta g\right)\right]}{\left(d+\Delta d\right)}\left[F\right]$$

and:

(10) $$S=\frac{\Delta C}{g}=\frac{n{ɛ}_{0}}{\left(d+\Delta d\right)}\left\{\frac{l}{2}\left[\left(l+2l^{\prime }\right)\theta \right]\right\}\left[F/g\right]$$

where Δ*t* is the variation range of device thickness (±2 μm), Δ*g* is the variation range of the vertical gap (±1 μm), and Δ*d* is the variation range of the gap between interdigitated comb electrode (± 0.8 μm). The simulation result of the nominal capacitance variation and the mechanical sensitivity variation is shown in Figure 8 (a,b), respectively. The nominal capacitance varies in the range of 49.8 pF∼24.5 pF, and this variation can cause a large offset variation, which results in a limitation of a sensor dynamic range. Also, the mechanical sensitivity varies in the range of 16.6 fF/g∼25.3 fF/g, and this can results in a large gain variation. Moreover, considering the parasitic capacitance variations after packaging, it is highly desired to design the compensation scheme at the analog front-end of a CMOS capacitive readout circuit.

**Figure 9.**
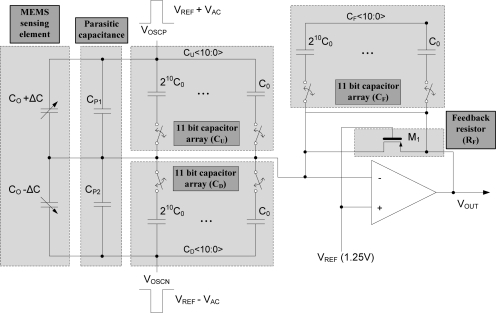
Schematic of analog front-end capacitive sensing circuit.

The proposed schematic of the capacitive analog front-end is shown in Figure 9. The output voltage of the front-end charge amplifier is derived as:

(11) $$V_{\text{OUT}}(s)=V_{\text{REF}}-\frac{sR_{F}}{sR_{F}C_{F}+1}\left[2\Delta C+\left(C_{P1}-C_{P2}\right)+\left(C_{U}-C_{D}\right)\right]V_{\text{AC}}(s)$$

where *V_OUT_* is the output voltage, *V_REF_* is the reference voltage (1.25 V), *R_F_* is the feedback resistor implemented by gate-biased transistor M_1_, *V_AC_* is the modulation clock, Δ*C* is the capacitance change of the MEMS sensing element, *s* is the chopping frequency, *C_P1_*, *C_P2_* are the parasitic capacitances, and *C_U_*, *C_D_*, *C_F_* are the 11 bit trimmable capacitor arrays. The output offset variation due to the parasitic capacitance, *C_P1_* and *C_P2_*, can be adjusted by trimming the *C_U_* and *C_D_* arrays. The minimum step of the trimmable capacitance (*C_0_*) is designed to be 5.374 fF and the maximum trimmable range is 11.002 pF.

In this capacitive interface circuit design, a continuous-time chopper stabilized sensing scheme is adopted. Since the capacitive circuit is implemented with a large feedback resistor (*R_F_* > 10 MΩ) and high chopping frequency (*s* = 500 kHz), *sRF* » 1 and Equation (11) can be simplified to:

(12) $$V_{\text{OUT}}(s)=V_{\text{REF}}-\frac{2\Delta C+\left(C_{P1}-C_{P2}\right)+\left(C_{U}-C_{D}\right)}{C_{F}}V_{\text{AC}}(s)$$

Thus, by trimming the *C_F_* capacitor array, the variation of analog front-end gain can be easily compensated.

## 5. Fabrication

### 5.1. Fabrication Flow

The MEMS sensing element is fabricated using the ESBM process. The ESBM process allow the fabrication of both upper and lower vertical comb gaps using only two photomasks and four DRIE steps, to achieve differential sensing. The differential sensing scheme results in a highly sensitive sensing element. Also, using the ESBM process, it is possible to fabricate a structure with an inherent high-aspect-ratio with a large sacrificial gap and a structure free from the footing phenomenon. A large sacrificial gap is required to minimize the disadvantages of a large parasitic capacitance, which results in higher gain and reduction in input-referred circuit noise. In addition, the large sacrificial gap has an advantage in protecting the suspended proof mass of the MEMS sensing element from drops and impacts. The WLHP process is employed by the glass-to-silicon anodic bonding to protect the MEMS sensing element and to achieve the reliability of the microsystem.

**Figure 10.**
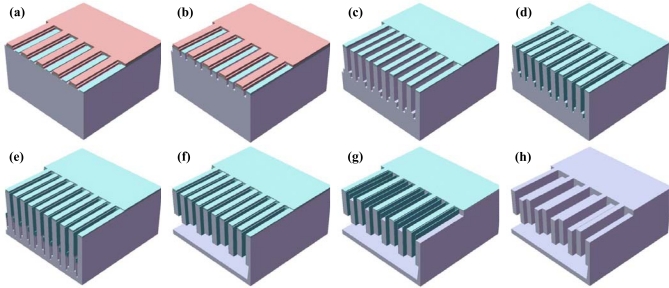
**(a)** 2nd photolighography & TEOS etch. **(b)** 1st DRIE. **(c)** 2nd DRIE. **(d)** Thermal oxidation & bottom etch. **(e)** 3rd DRIE. **(f)** Anisotropic wet etching. **(g)** Thinner TEOS etch & 4th DRIE. **(h)** Passivation layer removal. The ESBM fabrication process flow.

Process flow of the ESBM process is shown in Figure 10 (a)∼(h). A (111)-oriented SOI wafer with device layer of 65 μm is used to fabricate the sensing element. After cleaning of the SOI wafer, tetra-ethyl-ortho-silicate (TEOS) hard mask is fabricated by TEOS deposition, first photolithography and plasma etching. The second photolithography process is performed to slightly etch the top TEOS layer of the lower comb electrode, which turns out to be the stationary comb electrode [Figure 10(a)]. First a DRIE process is done to determine the lower vertical gap length [Figure 10(b)]. After stripping the photoresist, a second DRIE process is carried out to determine the structural thickness [Figure 10(c)]. As analyzed previously, the structural thickness of 25 μm gives the minimized noise performance characteristic for the MEMS sensing element. Also, this result provides a large sacrificial thickness in order to minimize the TNEA level. A thermal oxidation process is used for sidewall passivation, and TEOS etching is then used to open the bottom area [Figure 10(d)]. The thickness of the sacrificial layer is defined by third DRIE process [Figure 10(e)], and the anisotropic wet etching process using the tetra-methyl-ammonium-hydroxides (TMAH) is performed [Figure 10(f)]. After cleaning and drying, a plasma etching is done to etch the thinner layer of the TEOS hard mask to open the silicon surface. Then, a fourth DRIE process is performed to define the upper vertical gap length [see Figure 10(g)]. Finally, the passivation layer is removed using hydrofluoric acid (HF) [Figure 10(h)].

**Figure 11.**
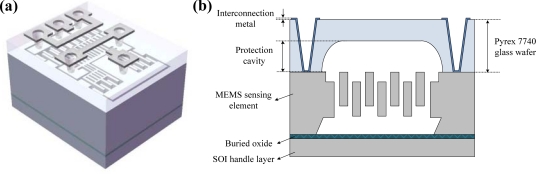
**(a)** Packaged device. **(b)** Cross-sectional view. Schematic diagram of packaged out-of-plane MEMS sensing element.

The WLHP process is employed by glass-to-silicon anodic bonding process. The Pyrex 7740 glass wafer with the thickness of 350 μm is used, and fabricated using HF glass wet etching. In order to neglect the damping inside the protection cavity, the thickness of fabricated protection cavity is fabricated to be 180 μm∼190 μm. After bonding, metal interconnections are fabricated by metal sputtering. A schematic diagram of the packaged MEMS sensing element and cross-sectional view are shown in Figure 11 (a,b), respectively.

### 5.2. Fabrication Result

**Figure 12.**
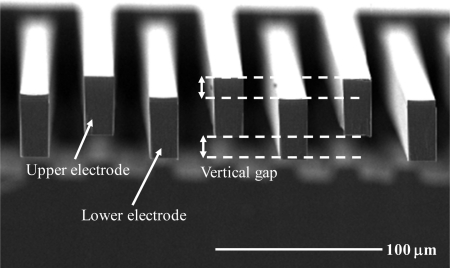
Test pattern fabricated using the ESBM process.

**Figure 13.**
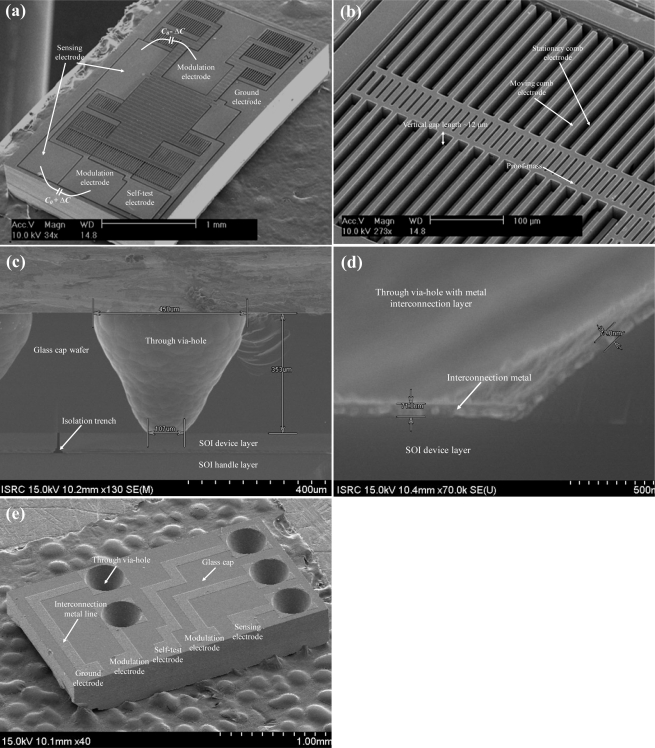
**(a)** Overall view of the fabricated silicon structure. **(b)** Magnified view of the fabricated vertical gap between interdigitated comb-fingers. **(c)** Cross-sectional view of the fabricated sensing element. **(d)** Magnified view of the metal interconnection layer and through via-hole. **(e)** Hermetic packaged MEMS sensing element. Fabricated MEMS out-of-plane microaccelerometer.

In Figure 12, a test pattern fabricated by ESBM process is shown. The upper and lower vertical gap is well fabricated, and as mentioned above, etch depth are defined by individual DRIE steps. The fabrication result of the overall MEMS sensing element with the vertical gap between the parallel comb electrodes is shown in Figure 13(a,b), respectively. The measured length of the vertical gap is 12 μm. The cross-sectional view of the packaged device, the cross-sectional view of the metal interconnection layer, and the overall view of the WLHP MEMS microaccelerometer are shown in Figure 13 (c,d,e), respectively.

## 6. Performance Evaluation

**Figure 14.**
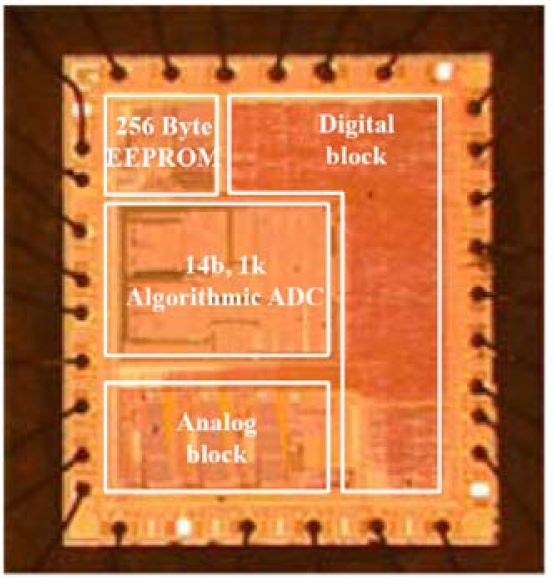
Microscope image of CMOS fabricated capacitive readout circuit.

The fabrication result of CMOS capacitive readout circuit is shown in Figure 14. The circuit is fabricated using the SMIC 0.35 μm process with 256 byte EEPROM. The die size is 2.01 mm × 2.36 mm.

**Figure 15.**
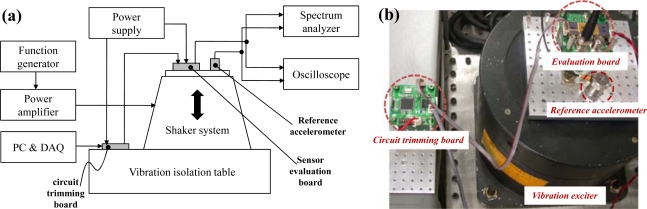
**(a)** Block diagram of experimental setup. **(b)** Photograph of experimental setup. Experimental setup.

The block diagram and photograph of the experimental setup are shown in Figure 15 (a,b), respectively. The two-chip implemented microsystem is mounted on a sensor evaluation board. The circuit trimming board is used to calibrate the offset and gain of the output signal. After trimming is done, the register value is saved in the 256 byte EEPROM. The B&K 8305 (Brüel & Kjær, Denmark) reference accelerometer is used to compare the output signal with the implemented microsystem. The Agilent 33250A (Agilent Technologies, Inc., United States) function generator is used to apply the acceleration source, and the accurate acceleration is generated to the microsystem using the B&K 4808 (Brüel & Kjær, Denmark) vibration exciter.

Before measuring the performance characteristics of the out-of-plane microaccelerometer system, the offset and gain calibration is carried out. From Figure 1, the final system output, *V_O_*, can be expressed as:

(13) $$V_{O}=V_{\text{REF}}-\left(\frac{2\Delta C+\left(C_{P1}-C_{P2}\right)+\left(C_{U}-C_{D}\right)}{C_{F}}+V_{\text{OFFSET}}\right)\cdot V_{I}\cdot A_{\text{GAIN}}$$

where *V_REF_* is the reference voltage (1.25 V), Δ*C* is the capacitance variation of the MEMS sensing element from applied external acceleration, *C_P1_* and *C_P2_* are the parasitic capacitances which is known to be in several pF ranges [7,8], *C_U_* and *C_D_* are the 11 bit capacitor arrays for parasitic cancellation, *C_F_* is a the 11 bit capacitor array for gain calibration, *V_OFFSET_* is the data register value of 9 bit current-mode DAC used for offset calibration, *V_I_* is the initial voltage when assuming ideal operational amplifier, and *A_GAIN_* is the data register value of 10 bit PGA of the output amplifier. With no external acceleration applied to the microsystem, the difference between the parasitic capacitances, *C_P1_* – *C_P2_*, can be derived by measuring *V_O_* and reading the data registers of *C_U_*, *C_D_*, *C_F_*, *V_OFFSET_*, and *A_GAIN_*. By trimming *C_U_* – *C_D_* equal to *C_P1_* – *C_P2_*, Equation (13) can be expressed as:

(14) $$V_{O}=V_{\text{REF}}-\left(\frac{2\Delta C}{C_{F}}+V_{\text{OFFSET}}\right)\cdot V_{1}\cdot A_{\text{GAIN}}$$

where the offset variation due to the parasitic capacitance is cancelled out. Then the fine tuning of the gain and offset can be done by trimming *C_F_* array, *A_GAIN_* and *V_OFFSET_*. The trimming range of the capacitor arrays (*C_U_*, *C_D_*, *C_F_*) is from 5.374 fF to 11.002 pF, *A_GAIN_* ranges from 2 V/V to 18 V/V, and *V_OFFSET_* ranges from −150 mV to +150 mV. The experiment is evaluated for three samples after calibration of the offset and gain. The samples are selected from center (sample #1), top (sample #2) and bottom (sample #3) side of the wafer to examine the die-to-die variation.

**Figure 16.**
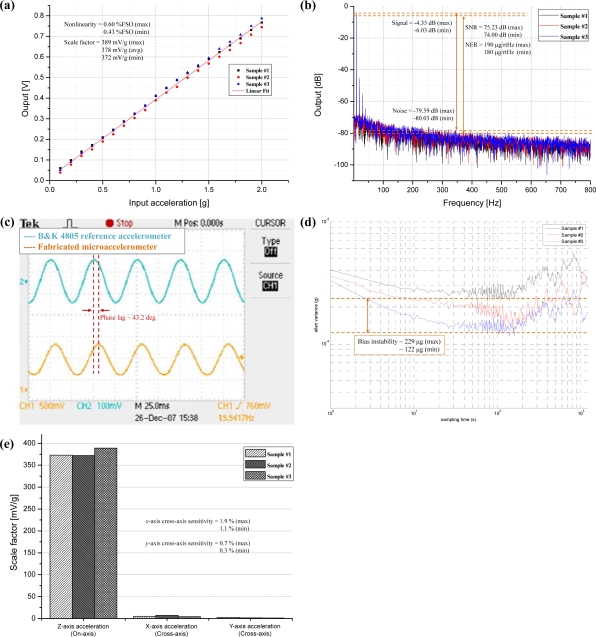
**(a)** Input-output characteristics at 10 Hz input acceleration. **(b)** Output spectrum at 10 Hz, 1 g input acceleration. **(c)** Time-domain output of Sample #1 at 20 Hz, 1g input acceleration. **(d)** Bias instability (Root-Allan variance plot). **(e)** Cross-axis sensitivity. Performance evaluations of out-of-plane microaccelerometer system.

The performance characteristic results are shown in Figure 16(a)∼(e). The input-output characteristic of the out-of-plane microaccelerometer system at 10 Hz input acceleration is shown in Figure 16(a). The input range and the average scale factor are measured to be ±2 g and 378 mV/g, respectively. The maximum output non-linearity is calculated to be 0.6% FSO. To measure the signal-to-noise ratio (SNR) and noise equivalent resolution (NER), the input acceleration of 10 Hz, 1 g sinusoidal wave is applied and the output spectrum is plotted [Figure 16(b)]. The thermal noise level is measured to be in the range from −80.03 dB to −79.59 dB and signal level is measured to be in the range from −6.03 dB to −4.35 dB. The maximum SNR is measured from sample #3 to be 75.23 dB, and minimum SNR is measured from sample #1 to be 74.00 dB. Therefore, the NER is calculated to be in the range from 180 μg/rtHz to 190 μg/rtHz. The measured noise floor of the each sample is 180 μg/rtHz, 183 μg/rtHz, and 190 μg/rtHz, which is higher than the result intended by the design. However, the similar results are shown between three samples, which reveal that the robustness to fabrication variations can be achieved by the proposed design method. Figure 16 (c) is the time-domain output of the microsystem with the input acceleration of 20 Hz, 1g sinusoidal wave. A phase lag of about 43.2 degree is observed. This phase lag is mainly occurred due to the low pass filter at the output stage of the fabricated CMOS capacitive readout circuit. In order to evaluate the bias instability, the root-Allan variance method [28] is adopted.

The root-Allan variance (*σ_total_*) can be simplified using the geometric mean of the velocity random walk (*σ_VRW_*) and the bias instability (*σ_BiasInst_*), expressed as:

(15) $$\sigma_{\text{total}}^{2}(\tau )=\sigma_{\text{VRW}}^{2}(\tau )+\sigma_{\text{BiasInst}}^{2}(\tau )=\frac{N^{2}}{\tau }+\frac{2\text{ln}2}{\pi }B^{2}$$

where *N*, *B* and *τ* is the velocity random walk coefficient, the bias instability coefficient, and the sampling time, respectively. Figure 16 (d) shows the root-Allan variance plots of the three samples. The bias instability is measured to have a value between 122 μg and 229 μg. The in-plane (*x*-axis and *y*-axis) cross-axis sensitivity is plotted in Figure 16 (e). Maximum value of *x*- and *y*-axis cross-axis sensitivity is measured to be 1.9% and 0.7%, respectively.

## 7. Conclusions

In this paper, an optimal and robust design method to implement a two-chip out-of-plane microsystem consisting of a MEMS chip for sensing the external acceleration and a CMOS chip for signal processing is presented. An optimized design method to determine the device thickness, the sacrificial gap, and the vertical gap length of the MEMS sensing element is applied to minimize the fundamental noise level and also to achieve the robustness to the fabrication variations. The MEMS sensing element is fabricated by the ESBM process to have a vertical differential sensing, and the WLHP process is performed so as to achieve the high reliability of the microsystem. In order to cancel out the offset and gain variations due to parasitic capacitance and to minimize the die-to-die variation due to fabrication mismatches, a digitally trimmable architecture consisting of the 11 bit capacitor array is adopted in CMOS capacitive readout circuit.

The summarized performance specifications are listed in Table 2. The out-of-plane microaccelerometer has the scale factor of 372 mV/g ∼ 389 mV/g, the output nonlinearity of 0.43% FSO∼ 0.60% FSO, the input range of ± 2 g and the bias instability of 122 μg∼229 μg. The SNR and the NER are measured to be 74.00 dB∼75.23 dB and 180 μg/rtHz∼190 μg/rtHz, respectively. The *x*- and *y*-axis cross-axis sensitivity is measured to be 1.1%∼1.9% and 0.3%∼0.7% of the out-of-plane sensitivity, respectively. The results show that the optimal and robust design method for the MEMS sensing element and the highly trimmable capacity of the CMOS capacitive readout circuit are suitable to enhance the die-to-die uniformity of the packaged microsystem without compromising the performance characteristics.

## Figures and Tables

**Table 1.** Summarized design parameters of the MEMS sensing element.

**Parameter**	**Symbol**	**Value**

Chip size		3.4 mm × 2.0 mm
Device layer thickness of (111) SOI wafer	*t_0_*	65 μm

**Torsional spring**		

Width	*w_spring_*	4 μm
Length	*l_spring_*	150 μm
Thickness	*t*	25 μm (±2 μm)
Number of springs	*n_spring_*	2
Torsion angle at 1 g input (at *t* = 25 μm)	*θ*	2.59 × 10^−4^ rad

**Comb finger**		

Width	*w*	5 μm
Length	*l_comb_*	210 μm
Thickness	*t*	25 μm (±2 μm)
Comb overlap length	*l*	200 μm
Vertical gap length	*g*	13 μm (±1 μm)
Gap between comb finger	*d*	4 μm
Effective distance from center to comb finger	*l’*	8.13 × 10^−4^ m
Number of combs	*n*	254

**MEMS structure**		

Area size	-	9.72 × 10^−6^ m^2^
Area of asymmetric part	-	2.74 × 10^−7^ m^2^
Center of mass	*x_cm_*	5.58 × 10^−4^ m
Thickness	*t*	25 μm (±2 μm)
Device length	*a*	2.53 mm
Device width	*b*	1.388 mm
1st mode natural frequency (at *t* = 25 μm)	*ω_n_*	598 Hz

**Table 2.** Summarized performance characteristics.

	**Sample #1**	**Sample #2**	**Sample #3**

**Scale factor**	373 mV/g	372 mV/g	389 mV/g
**Non-linearity**	0.60%FSO	0.59%FSO	0.43%FSO
**SNR**	74.00 dB	74.74 dB	75.23 dB
**NER**	180 μg/rtHz	183 μg/rtHz	190 μg/rtHz
**Bias instability**	229 μg	148 μg	122 μg
**Cross-axis sensitivity (x-axis)**	1.3%	1.9%	1.1%
**Cross-axis sensitivity (y-axis)**	0.7%	0.4%	0.3%
